# Synthesis and physicochemical, DFT, thermal and DNA-binding analysis of a new pentadentate N_3_S_2_ Schiff base ligand and its [CuN_3_S_2_]^2+^ complexes

**DOI:** 10.1039/d0ra04323k

**Published:** 2020-06-10

**Authors:** Ismail Warad, Hadeel Suboh, Nabil Al-Zaqri, Ali Alsalme, Fahad A. Alharthi, Meshari M. Aljohani, Abdelkader Zarrouk

**Affiliations:** Department of Chemistry and Earth Sciences, Qatar University PO Box 2713 Doha Qatar ismail.warad@qu.edu.qa; Department of Chemistry, Science College, An-Najah National University P.O. Box 7 Nablus Palestine; Department of Chemistry, College of Science, King Saud University P.O. Box 2455 Riyadh 11451 Saudi Arabia; Department of Chemistry, College of Science, Ibb University P.O. Box 70270 Ibb Yemen; Department of Chemistry, Faculty of Science, University of Tabuk Tabuk-71491 Saudi Arabia; Laboratory of Materials, Nanotechnology and Environment, Faculty of Sciences, Mohammed V University Av. Ibn Battouta, Box 1014 Agdal-Rabat Morocco

## Abstract

A new N_3_S_2_ pentadentate Schiff base ligand derived from 5-bromothiophene-2-carbaldehyde, (*E*)-*N*1-((5-bromothiophen-2-yl)methylene)-*N*2-(2-((*E*)-((5-bromothiophen-2-yl)-methylene amino) ethyl ethane-1,2-diamine, is prepared. The ligand and its complexes are subjected to extensive physical and theoretical analyses and the results are consistent with their predicted compositions. Dicationic Cu(ii) complexes ([CuN_3_S_2_]X_2_) with a coordination number of 5 are proposed on the basis of the spectral data with N_3_S_2_ serving as a pentadentate ligand. The prepared complexes display a square pyramidal geometry around the Cu(ii) center. TG shows different thermal behavior for the N_3_S_2_ ligand and its complexes. Solvatochromism of the complexes is promoted by the polarity of the solvent used. A one-electron transfer Cu(ii)/Cu(i) reversible redox reaction is promoted by CV. SEM and EDS of the free ligand and its complexes support the morphology and composition changes observed upon the complexation of Cu(ii). As an outstanding goal to develop anticancer new metal chemotherapy, preliminary studies of the binding of the desired complexes with DNA were carried out, as it is through judging the strength of interactions that a future drug can be designed and synthesized. The viscosity and absorption results obtained for complex 1 indicated its enhanced CT-DNA binding properties as compared to those of complex 2 with *K*_b_ values of 3.2 × 10^5^ and 2.5 × 10^5^ M^−1^, respectively.

## Abstract 2

A new N_3_S_2_ pentadentate Schiff base ligand derived from 5-bromothiophene-2-carbaldehyde, (*E*)-*N*1-((5-bromothiophen-2-yl)methylene)-*N*2-(2-((*E*)-((5-bromothiophen-2-yl)-methylene amino) ethyl ethane-1,2-diamine, is prepared.

## Graphical abstract

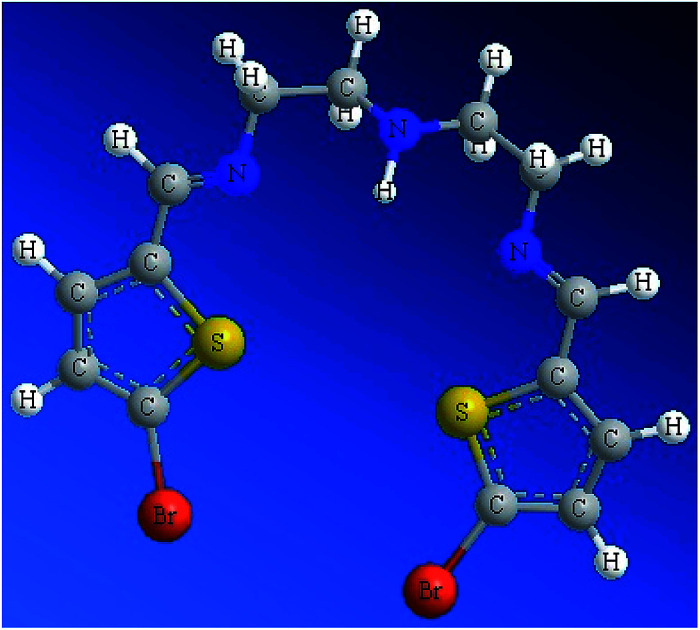

## 1. Background

Multidentate ligands are frequently used in organometallic chemistry because of their poly-bonding ability to coordinate with several metal ions.^1–3^ Consequently, systems containing multidentate ligands are critical towards the success of coordination studies.^3^ One of the most famous multidentate ligands are Schiff bases, which have attracted the attention of researchers due to their ability to coordinate to metals *via* several sites, which can stabilize novel structures around the metal center.^3–8^ In general, Schiff base-transition metal complexes that have been extensively investigated because the Schiff base ligand can coordinate with metal ion centers *via* one or more sites leading to the synthesis of several types of complex with different metal centers and stereochemistry, and a broad range of applications.^8–10^ Recently, several reports have shown that these ligands and their complexes can be used toward the design of new drug candidates exhibiting anti-cancer, enzyme inhibition, anti-malarial, antifungal, antibacterial, and anti-inflammatory activity.^8–16^

Copper is essential in the human body and plays a critical role in biological processes involving electron transfer reactions. In fact, Cu(ii) complexes bearing {S, O, N} donor chelating ligands are excellent anti-cancer agents because of their strong DNA binding ability.^4–10^ Due to the highly selective permeability of copper(ii) ions through the cell membrane of cancer cells, copper is considered to be one of the most effective anti-tumor agents with low cost and few side effects.^7–16^ Thus, various complexes bearing several types of ligand have been prepared and evaluated against cancer cells.^12–24^ Pentadentate Schiff base ligands have received less attention as compared to mono-, di-, tri-, and tetradentate ligands due to the difficulty in their synthesis and unexpected multimode coordination behavior.^15–24^ In view of the several coordination modes exhibited by pentadentate N_3_S_2_ ligands derived from thiophene, their interesting structures, and the CT-DNA binding affinity of their Cu(ii) complexes, we herein report two mononuclear copper(ii) complexes obtained using a new pentadentate N_3_S_2_ ligand. The CT-DNA binding affinity of their corresponding Cu(ii) complexes is also evaluated.

## 2. Experimental

### 2.1. Material and instrumentation

All chemicals and solvents were purchased from Sigma and used without any further purification. TLC was performed to evaluate the purity of the as-synthesized compounds when needed. Elemental analysis was carried out on an Elementar-Vario EL analyzer. TG/DTG curves were recorded on a Perkin-Elmer thermogravimetric analyzer. FT-IR spectroscopy was recorded on a Perkin-Elmer Spectrum 1000 FT-IR spectrometer. UV-Vis spectroscopy was recorded on a TU-1901double-beam UV-visible spectrophotometer. All electrochemical experiments were carried out at room temperature under an argon atmosphere using a three-electrode cell Voltalab 80 potentio-state PGZ402 equipped with a Pt-electrode (Metrohm, *A* = 0.0064 cm^2^) used as the working electrode and platinum wire spiral (£1 mm) with a diameter of 7 mm used as the counter electrode.

### 2.2. Synthesis of *N*-[(1*E*)-(5-bromothien-2-yl)methylene]-*N*′-(2-{[(1*E*)-(5-bromothien-2-yl)methylene]-amino}ethyl)ethane-1,2-diamine [N_3_S_2_]

5-Bromothiophene-2-carbaldehyde (0.026 mol) was added to diethylenetriamine (0.013 mol) in the absence of solvent and the resulting mixture was stirred for 30 min at RT until a viscous oil was formed. The temperature increased and the viscosity of the reaction mixture ensured the condensation reaction occurred. Dichloromethane (10 mL) was added to the reaction mixture and the resulting solution was sealed and stirred for 1 h. A colorless oily product was obtained after evaporation of the solvent.

Yield: 85%. The product was a colorless oil at RT. Molecular formula: C_14_H_15_Br_2_N_3_S_2_. ^1^H NMR (250 MHz, CDCl_3_) *δ* (ppm): 2.4 (t, 4H, –HNCH̲_2_CH_2_N=CH–), 3.9 (t, 4H, –HNCH_2_CH̲_2_N=CH–), 4.3 (broad s, 1H, HN only observed in free dissolved sample), 6.7 (d, 2H, thiophene), 7.6 (d, 2H, thiophene), 8.2 (s, 2H, –HC=N–). ^13^C NMR *δ* (ppm): 15.8 (s, 2C, –HNC̲H_2_CH_2_N=CH–), 61.1 (s, 2C, –HNCH_2_C̲H_2_N=CH–), 125.6, 130.0, 140.0, 143.0 (d, 8C, thiophene), 156.1 (s, 2C, –HC=N–). MS: *m*/*z* = 446.2 [M^+^]. UV-Vis (EtOH) (nm): 240 (sh), 280. IR (cm^−1^): *ν* = 3320 (N–H), 3020 (C–H thiophene), 2960–2770 (C–H aliphatic), 1675 (C=N).

### 2.3. Synthesis of complexes 1–2

A solution of N_3_S_2_ (0.18 mmol) in EtOH (5 mL) was added to the Cu(ii) salt (0.17 mmol) dissolved in freshly distilled EtOH (20 mL). The color of the reaction mixture changed from brown to blue and the temperature increased upon the addition of the ligand solution. The product complex is poorly soluble in EtOH and precipitates from the reaction mixture. A reduction in the volume of the reaction mixture under vacuo led to most of the blue Cu(ii) complex being precipitated, which was then filtered and washed with cooled EtOH and ethyl acetate.

#### 2.3.1. Complex 1

Yield: 90%. mp = 204 °C. MS: *m*/*z* = 507.2 [M^2+^]. Anal. (%): [C_14_H_15_Br_2_CuN_3_S_2_]Cl_2_, calculated: C, 28.81; H, 2.59; found C, 28.66; H, 2.45. Conductivity in water: 185 μS cm^−1^. IR (KBr, cm^−1^): *ν* = 3360 (H_2_O), 3250 (N–H), 3010 (C–H thiophene), 2930 (C–H), 1655 (C=N), 1590 (N–H), 1150 (N–C), 520 (Cu–N). UV-Vis (water, nm): 250 (2.2 × 10^4^ M^−1^ L^−1^) and 615 (2.8 × 10^2^ M^−1^ L^−1^).

#### 2.3.2. Complex 2

Yield: 92%. mp = 220 °C. MS: *m*/*z* 507.2 [M^2+^]. Anal. (%): [C_14_H_15_Br_2_CuN_3_S_2_]Br_2_, calculated: C, 25.00; H, 2.25; found C, 24.88; H, 2.1. Conductivity in water: 210 μS cm^−1^. IR (KBr, cm^−1^): *ν* = 3360 (H_2_O), 3280 (N–H), 3030 (C–H thiophene), 2910 (C–H), 1658 (C=N), 1580 (N–H), 1180 (C–N), 510 (Cu–N). UV-Vis (water, nm): 260 (2.3 × 10^4^ M^−1^ L^−1^) and 625 (2.2 × 10^2^ M^−1^ L^−1^).

### 2.4. DNA binding experiments

The experimental titration absorption spectra were recorded in Tris–HCl buffer (5 mM Tris–HCl/50 mM NaCl buffer at pH 7.2) using a Cu(ii) complex concentration of 5.0 × 10^−5^ M (complex 1) and 1.0 × 10^−5^ M (complex 2) throughout the experiment. The CT-DNA concentrations were varied between 0 and 5.0 × 10^−5^ M (complex 1) and 0 and 1.0 ×10^−3^ M (complex 2), maintaining the total mixture volume constant at 10.0 mL. The resulting mixed solution of Cu(ii)/CT-DNA was allowed to equilibrate for 10 min at RT for each experiment prior to carrying out the absorption measurement.^35–39^

### 2.5. Viscosity experiments

Viscosity experiments were performed on a Ubbelodhe viscometer at 25.0 (±0.1) °C. The flow time was measured using a stopwatch with different concentrations of the complexes (0, 2.5 × 10^−5^, 6.25 × 10^−5^, 8.75 × 10^−4^, 1.12 × 10^−4^, and 1.37 × 10^−4^ M) and a fixed concentration of DNA = 5.0 × 10^−4^ M in Tris–HCl buffer. Each sample was measured in triplicate and the average flow time was calculated. Data are presented as (*η*/*η*^o^)^1/3^*versus* the binding ratio [Cu]/[DNA],^35–41^ where *η* is the viscosity of DNA in the presence of the complex and *η*^o^ is the viscosity of the pure DNA solution.

## 3. Results and discussion

### 3.1. Synthesis

**Scheme 1:**
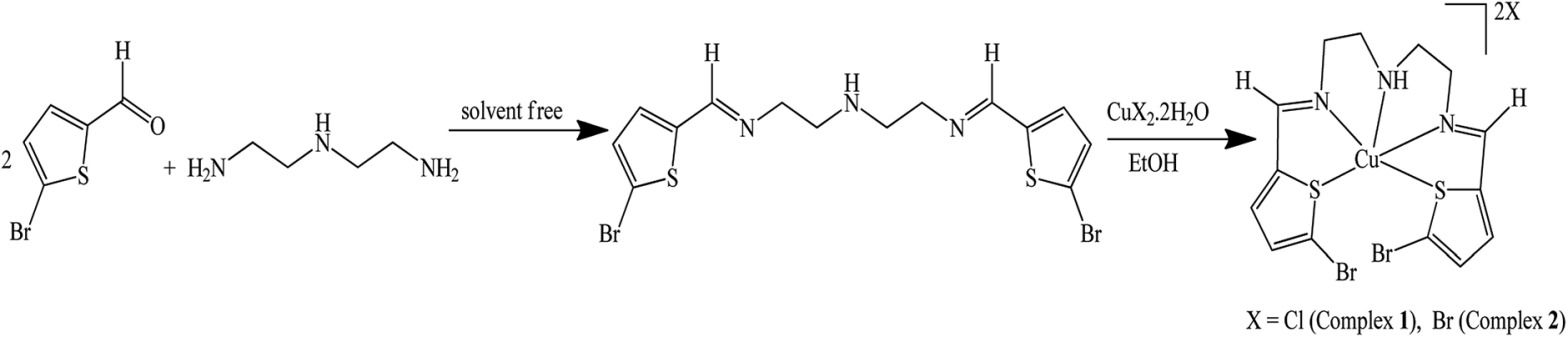
Synthesis of the N_3_S_2_ ligand and its Cu(ii) complexes.

A solvent-free condensation reaction using a 2 : 1 molar ratio of 5-bromothiophene-2-carbaldehyde and diethylenetriamine under an air atmosphere rapidly affords a new pentadentate Schiff base ligand, (*E*)-*N*1-((5-bromothiophen-2-yl)methylene)-*N*2-(2-((*E*)-((5-bromothiophen-2-yl)methylene)amino)ethyl)ethane-1,2-diamine (N_3_S_2_), in good yield (Scheme 1).

The desired Cu(ii) complexes were prepared by mixing an equivalent amount of N_3_S_2_ with the hydrated CuX_2_ salt in EtOH at RT under an atmosphere. The preparation of complexes 1 and 2 was confirmed by the color and temperature changes observed in the reaction. The addition of the colorless ligand solution to the copper salt solution was accompanied by a distinct color change from brown to blue and the isolated complexes were characterized using spectroscopic, electrochemical, and thermal analysis.

The complexes are very soluble in coordinating-solvents such as water, DMSO, and DMF, and poorly soluble in alcohols including ethanol, which strongly indicates the complexes are ionic. The molar conductivity of the aqueous complex solutions was 185–210 μS cm^−1^, which is within the range observed for a [1 : 1] electrolyte.

The structures of the N_3_S_2_ ligand and its complexes were analyzed using EA, MS, FT-IR, CV, NMR, UV-Vis, SEM, EDS, and TG/DTG. DFT calculations on the free ligand were performed using Gaussian 09 software. The analysis of the complexes revealed the construction of [Cu:N_3_S_2_]X_2_-type square pyramid complexes. The mass spectra, conductance, and water solubility of the as-prepared complexes supported their dicationic mononuclear salt nature, as illustrated in Scheme 1.

### 3.2. Optimized structure of N_3_S_2_

**Fig. 1:**
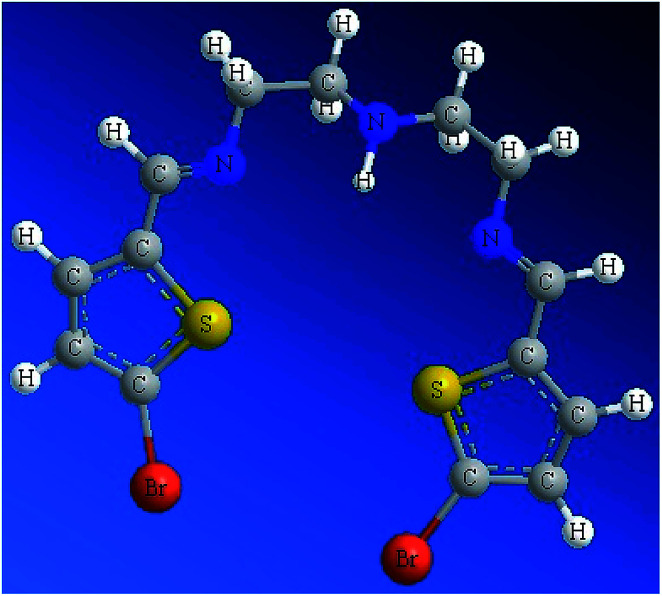
. DFT-optimized structure of the N3S2 ligand.

Since the ligand is an oil at RT, it was not possible to collect a stable and suitable crystal for X-ray analysis. Subsequently, the structure of the N_2_S_2_ ligand was optimized at the DFT-B3LYP level of theory, as depicted in Fig. 1. The optimized molecular structure of the N_2_S_2_ ligand revealed the (*E*,*E*)-isomer of both C=N groups was the kinetically favored isomer with the least internal strict repulsion effect, which forces the S-heterocyclic rings to be in the same plane creating a semi-vacant site center suitable to be occupied by metal ions. The presence of two aromatic rings conjugated to the two C=N groups results in the increased acidity of the N–H group in the ligand.^4–10^

### 3.3. MS and elemental analysis

**Fig. 2:**
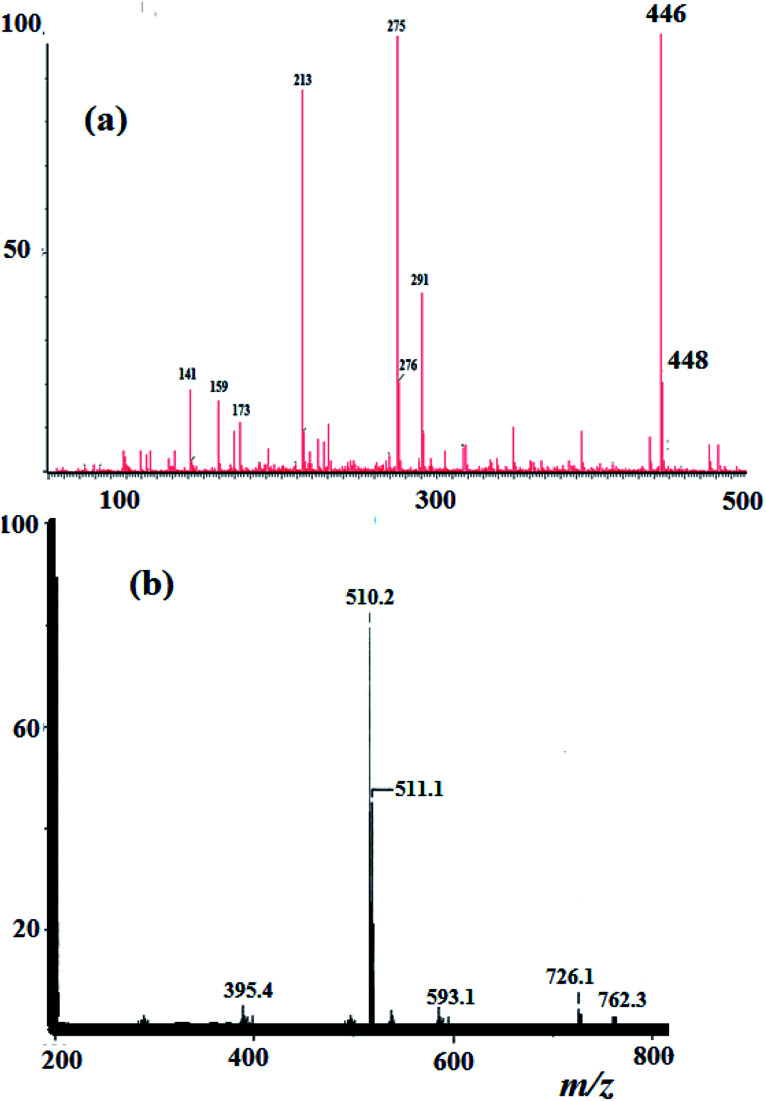
(a) EI-MS spectrum recorded for the N_3_S_2_ ligand and (b) ESI-MS spectrum of complex 2.

Elemental analysis result of the N3S2 ligand and its Cu(ii) complexes was in agreement with their proposed molecular formulae. For the N_3_S_2_ ligand: C_14_H_15_Br_2_N_3_S_2_, calcd. C, 37.43; H, 3.37%; found: C, 37.25; H, 3.21%. EI-MS of the ligand was in accordance with its assigned structure: *m*/*z* = 446.2 [M^+^] and 448.2 [M^+^ + 2], (theoretical *m*/*z* = 446.9), as shown in Fig. 2a.

The ESI-MS data obtained for the complexes are consistent with their proposed formula and support their monomeric dicationic structure. The theoretical *m*/*z* value of complex 2 is 509.2 [M^+^] and was observed experimentally with molecular ion peaks at *m*/*z* = 510.2 [M^+^ + 1] and 511.1 [M^+^ + 2], which confirm its dicationic mononuclear structure and molecular formula, as shown in Fig. 2b.

### 3.4. ^1^H and ^13^C-NMR spectra of the N_3_S_2_ ligand

**Fig. 3:**
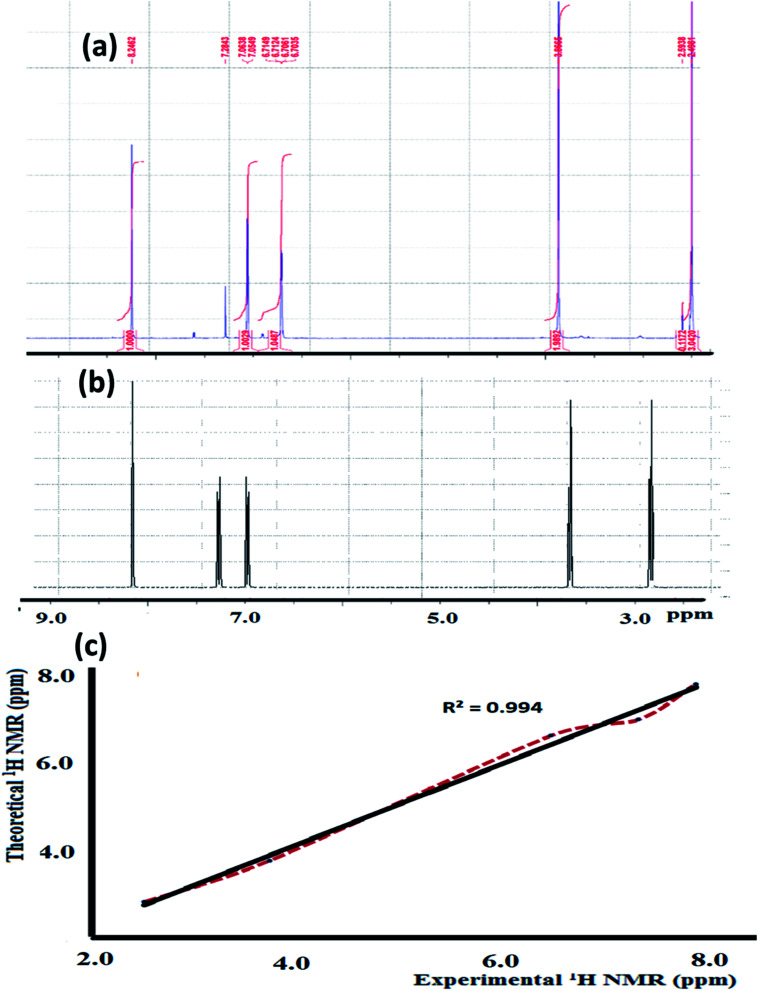
(a) Experimental and (b) theoretical ^1^H NMR spectrum of the N_3_S_2_ ligand recorded in CDCl_3_ at RT and (c) comparison of the experimental and theoretical spectra.

The experimental ^1^H-NMR spectrum of the N_3_S_2_ ligand was recorded in CDCl_3_ and shown in Fig. 3a; the theoretical spectrum is depicted in Fig. 3b and a comparison between the theoretical and experimental ^1^H NMR spectra is shown in Fig. 3c. The ^1^H NMR spectrum shows two sharp triplet signals at *δ* = 2.4 and 2.9 ppm corresponding to =NCH_2_CH̲_2_NHCH̲_2_CH_2_N= and =NCH̲_2_CH_2_NHCH_2_CH̲_2_N=, respectively. No signal for the NH proton was detected due to the rapid D/H exchange parallel to the CDCl_3_/CHCl_3_ singlet observed at *δ* = 7.2 ppm (the NH proton appears as a broad singlet peak at *δ* = 4.3 ppm in the freshly prepared NMR solution of the ligand), which is in consistent with the calculated acidity of the NH group. The thiophene protons were observed as two multiplet peaks at *δ* = 6.7 and 7.6 ppm, and the azomethine proton (N=CH) was detected as a singlet at *δ* = 8.2 ppm.

The theoretical ^1^H-NMR spectrum shows several peaks belonging to the aliphatic, thiophene, and azomethine protons in the N_3_S_2_ ligand, which are consistent with the experimental spectrum. For comparison, the theoretical ^1^H-NMR spectrum was plotted against the experimental one, as depicted in Fig. 3c. Fig. 3c shows the matched linear relationship between the theoretical and experimental ^1^H-NMR spectra, which reflects the excellent degree of agreement.

**Fig. 4:**
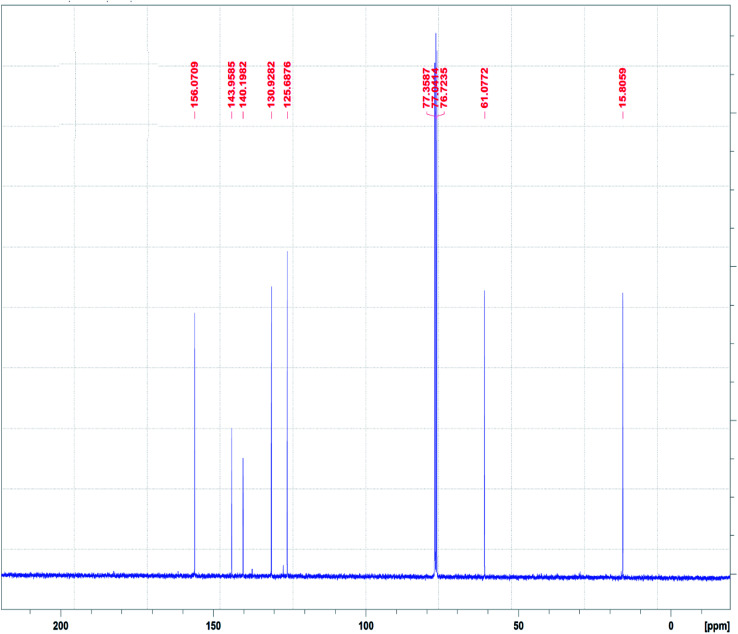
^13^C NMR spectrum of the N_3_S_2_ ligand recorded in CDCl_3_ at RT.

The ^13^C NMR spectrum of the N_3_S_2_ ligand is shown in Fig. 4. The ^13^C NMR spectrum exhibits two singlet peaks at *δ* = 15.0 (CH_2_NH) and 61.1 (CH_2_N=) ppm. The four aromatic carbon signals are observed at *δ* = 125.6, 130.0, 140.0, and 143.0 ppm, and the N=CH signal was observed at *δ* =156.0 ppm.

### 3.5. FT-IR and DFT-IR spectroscopy

**Fig. 5:**
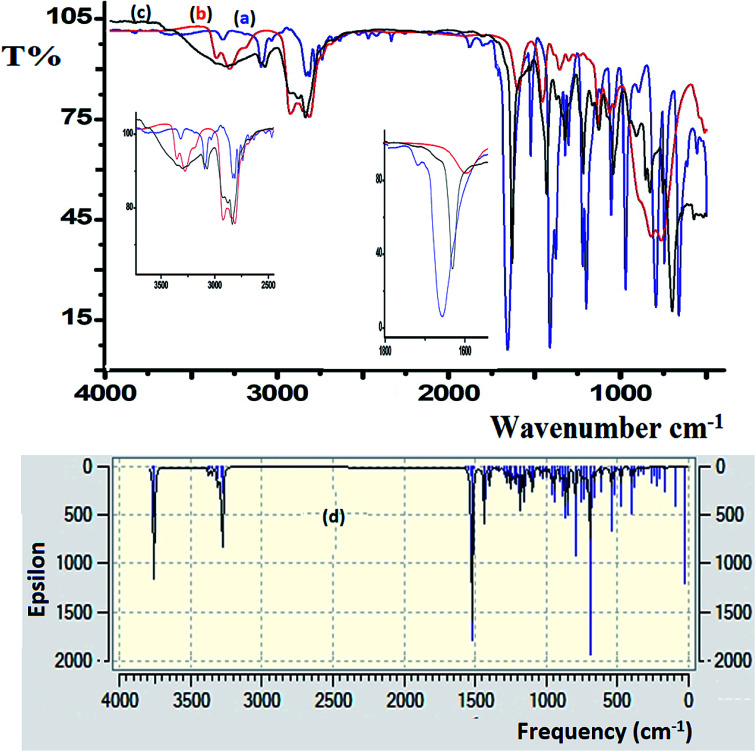
FT-IR spectra recorded for (a) 5-bromothiophene-2-carbaldehyde, (b) diethylenetriamine, and (c) the N_3_S_2_ ligand. (d) DFT-IR theoretical spectrum of the N_3_S_2_ ligand.

FT-IR spectroscopy was utilized to monitor the condensation reaction during the ligand formation step. The FT-IR spectra of the starting materials, 5-bromothiophene-2-carbaldehyde and *N*1-(2-aminoethyl)ethane-1,2-diamine, before and after the condensation reaction were recorded (Fig. 5). The formation of the ligand was confirmed by two major changes: (1) the primary N–H stretching vibration in diethylenetriamine observed at 3340 and 3270 cm^−1^ (Fig. 5a) is reduced to one single peak at 3240 cm^−1^ due to the formation of the secondary amine in the ligand (Fig. 5b); and (2) the stretching vibration belonging to the C=O group in carbaldehyde observed at 1742 cm^−1^ is shifted by ∼60 cm^−1^ due to the formation of the C=N– group (1688 cm^−1^) in the ligand (Fig. 5c).

DFT-IR calculations were also carried out for the free ligand, as shown in Fig. 5d. The experimental result was expected to be lower than the theoretically calculated value because the DFT-IR calculations were performed for a free molecule in the gaseous state, while the experimental spectrum was recorded in the solid state.^25–27^

The theoretical and experimental FT-IR spectra are illustrated in Fig. 5c and d for comparison, in which the vibrational frequencies and intensities were in agreement with each other.

**Fig. 6:**
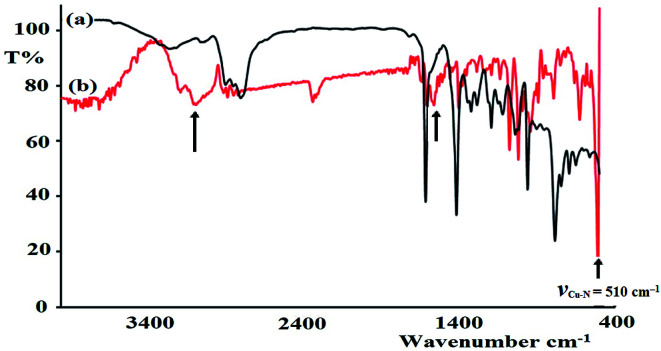
IR spectra recorded for the (a) N_3_S_2_ ligand and (b) complex 2.

The FT-IR spectra of the as-synthesized complexes are similar to that observed for the free ligand with slight shifts in the peak positions (Fig. 6). Fig. 6 illustrates the differences observed in the FT-IR spectra recorded for the ligand and complex 2. In complex 2, the water peak vibrations are observed at ∼3425 (*ν*_(O–H)_) and 1422 (*ν*_(bend)_) cm^−1^ indicating the presence of uncoordinated water molecules in the lattice of the complex and not in the ligand, since the complex is water soluble, but the ligand is not. The *ν*_(N–H)_ band observed at 3250 cm^−1^ in the complex was shifted to a lower wavenumber with high intensity compared to that of the N_3_S_2_ ligand (3320 cm^−1^), which may be attributed to the coordination of the NH group to the copper metal center. In addition, the *ν*_(C=N)_ vibration peak of the complex was shifted by ∼23 cm^−1^ (from 1688 to 1665 cm^−1^) due to the formation of the C=N → Cu(ii) bond. The most important observation in this FT-IR study was the presence of a sharp peak at 510 cm^−1^ in the complex spectrum due to the *ν*_(Cu–N)_ vibrations, which support the direct formation of the new N → Cu(ii) bonds.

### 3.6. Frontier molecular orbital calculations

**Fig. 7:**
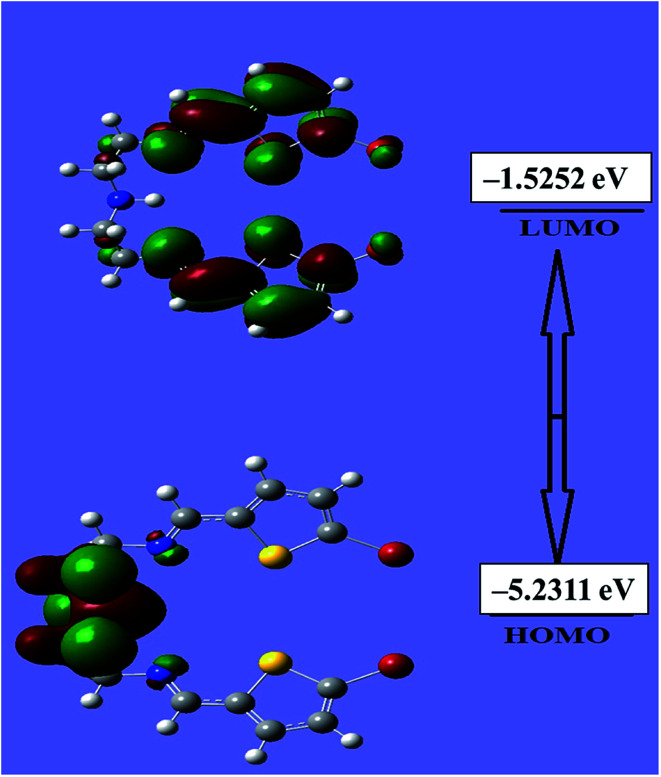
HOMO and LUMO of the N_3_S_2_ ligand.

An evaluation of the HOMO/LUMO energies is beneficial toward estimating the chemical behavior of the N_3_S_2_ ligand. The HOMO/LUMO energy gap controls many chemical reactivity descriptors, such as hardness, electrophilicity, quantum chemistry terms, chemical potential, electronegativity, and local reactivity.^25–28^ For example, the nucleophilicity of the ligand can be evaluated by its ability to donate electrons, which is associated with the HOMO energy level, while the electron affinity is characterized by the LUMO. Fig. 7 shows a schematic representation of the HOMO/LUMO orbitals in the gaseous phase. The HOMO is located at −5.2311 eV, while the LUMO is located at −1.5252 eV with an energy gap of 3.7059 eV. The calculated energy gap value reveals the ease of HOMO to LUMO electron excitation, which is reflected in the HOMO being the predominant molecular orbital and consistent with overall nature of the pentadentate ligand as a strong electron donor with a high degree of nucleophilicity. It is very easy for electrons to be excited from the ground to excited state with such a small energy gap. The HOMO/LUMO gap is related to the chemical reactivity or kinetic stability, since both have negative values. Consequently, the HOMO and LUMO decide the chemical stability of the ligand.^22–31^ Several parameters related to the HOMO/LUMO energy gap value have been theoretically calculated and are illustrated Table 1.

**Table 1:** Calculated energy values obtained using the B3LYP/3-21G level of theory

Basis set	B3LYP/3-21G
*E*_HOMO_	−5.2311
*E*_LUMO_	−1.5252
Chemical potential (*μ*)	−6.7563
Dipole moment	2.49140
Chemical hardness (*η*)	1.85255
Electronegativity (*X*)	6.75632

### 3.7. UV-Vis spectroscopy

**Fig. 8:**
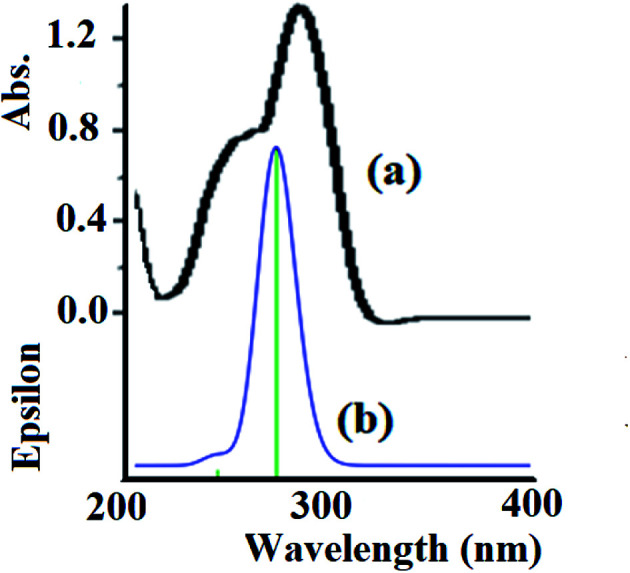
(a) Experimental UV-Vis spectrum of the N_3_S_2_ ligand recorded in ethanol at RT and (b) the TD DFT/B3LYP/3-311 theoretical spectrum calculated in the gaseous phase.

The electronic absorption behavior of the N_3_S_2_ ligand and its complexes were measured at RT in ethanol and water, respectively. The absorption bands observed for the ligand were also assigned using theoretical calculations at the TD-DFT/B3LYP/3-21 level of theory. The UV-Vis spectrum of the N_3_S_2_ ligand shows highly intense transitions at *λ*_max_ = 240 (sharp) and 280 nm (3.0 × 10^4^ M^−1^ L^−1^), which correspond to the π–π* transitions, as shown in Fig. 8a. The absorption maxima in the time-dependent DFT UV-Vis spectra were observed at 275 (sharp) and ∼1100 nm (broad, out of range >800 nm), as shown in Fig. 8b. The theoretical UV-Vis calculations of the molecular orbital geometry revealed the visible absorption maximum of the N_3_S_2_ ligand corresponds to the electron transition between the HOMO and LUMO. A good agreement between the theoretical-TD-DFT results and experimental UV-Vis spectra was observed and the slight ∼5 nm shift was attributed to the solvent effect.^26–30^

**Fig. 9:**
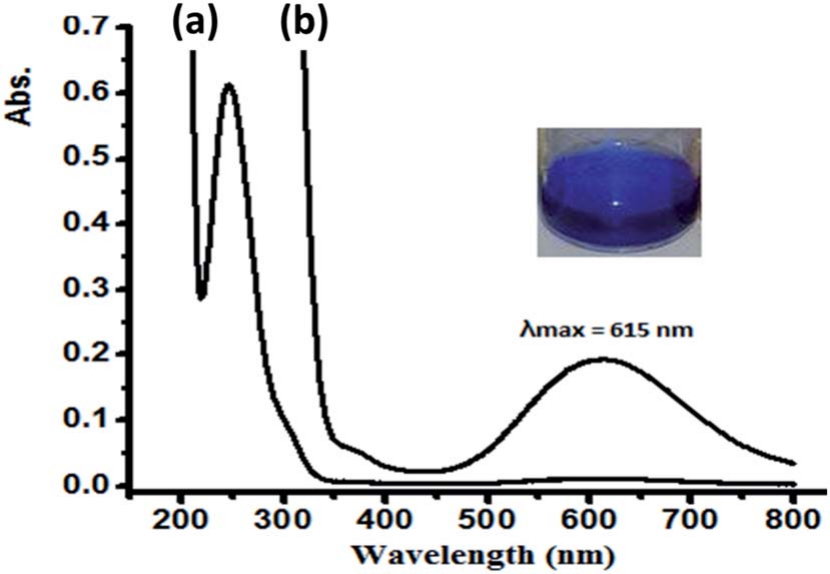
UV-Vis spectra recorded for complex 1 dissolved in water at RT at a concentration of (a) 5 × 10^−5^ M and (b) 5 × 10^−4^ M.

In water, complex 1 and 2 exhibit similar electronic behavior. The signals corresponding to the π–π* electron transition were shifted from 280 (free ligand) to ∼250 nm in the complexes due to the coordination of the ligand to the Cu(ii) center. In addition, broad bands in the visible region of 600–640 nm were observed upon complexation with the Cu(ii) center, which were not exhibited by the free ligand or CuX_2_ starting material. These bands were attributed to the blue color of the resulting N–Cu(ii) complexes. The blue color absorption was assigned to the d–d electron transition in the square pyramid geometry around the Cu(ii) complex center, as shown in Fig. 9 for complex 1.

### 3.8. Solvatochromism of complex 2

Water, EtOH, DMF, and DMSO were used to evaluate the solvatochromism phenomena observed due to the solubility of the dicationic complexes. The UV-Vis spectra of complex 2 recorded in the selected solvents exhibit a broad band at 600–800 nm. The complexes exhibited a significant positive *λ*_max_ shift upon increasing the polarity of the solvent due to the expected Jahn–Teller effect observed at the Cu(ii) center with a d^9^ electronic configuration and coordination number of 5.

**Fig. 10:**
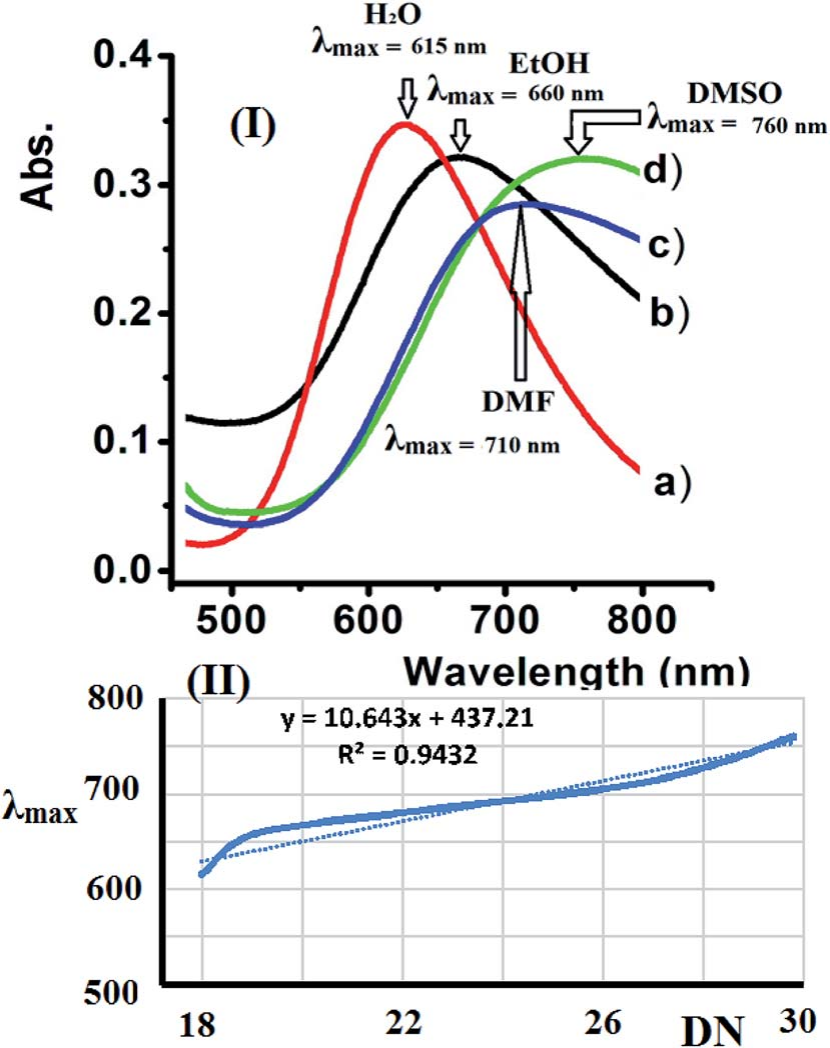
(I) Absorption spectra recorded for complex 2 dissolved in the selected solvents and (II) the dependence of the *λ*_max_ value of complex 2 on Gutmann's donor number (DN) for the solvents studied.

The UV-Vis spectra of complex 2 shift depending on the solvent's donor number polarity, as shown in Fig. 10a. Bathochromic shifts were recorded due to the direct coordination of the polar solvent molecules to the vacant sites of the five coordination Cu(ii) center with different strengths, which is in accordance with the mechanism of solvatochromism reported for this type of complex.^32–34^

Accordingly, the *λ*_max_ values observed for complex 2 in the different solvents studied increases linearly when Gutmann's donor number (DN) of the selected solvent increased. The linear trend in *λ*_max_ observed for complex 2 verses DN is shown in Fig. 10b.

### 3.9. Thermal analysis

**Fig. 11:**
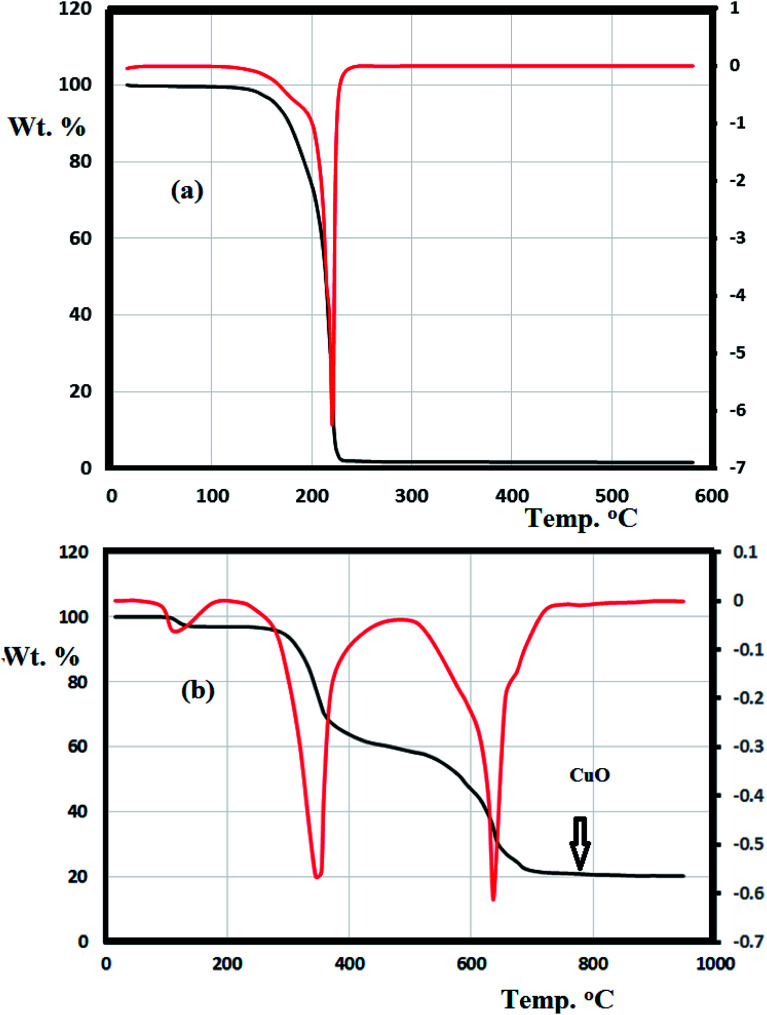
TG/DTG thermal curves obtained for (a) the N_3_S_2_ ligand and (b) complex 2.

The thermal behavior of the N_3_S_2_ ligand and complex 2 were investigated using TG/DTG under an open air atmosphere over a temperature range of 0–900 °C at a heating rate of 10 °C min^−1^ (Fig. 11).

Fig. 11a shows the TG curve obtained for the N_3_S_2_ ligand, which displays noticeable thermal stability up to 140 °C. Decomposition begins after 140 °C and ends at ∼260 °C. The ligand was totally decomposed into light gases including SO_2_, NO_2_, and CO_2_ in a broad step with ∼100% weight loss. No intermediate degradation steps, physical phonemes, or residues were recorded and the compound exhibited a simple one-step thermal decomposition mechanism.

Complexes 1 and 2 exhibit similar thermogravimetric behavior. The TG/DTG curve obtained for complex 2 shows three main steps (Fig. 11b). The first step (<100 °C) corresponds to the loss of uncoordinated water molecules in accordance with the FT-IR results. The second decomposition step at 280–450 °C (40% weight loss) was attributed to the decomposition of the ligand from the backbone of complex 2 to give CuBr_2_ as the final product. The third step starts from 460 °C and ends at 750 °C, which was attributed to the reaction between CuBr_2_ and O_2_ with the elimination of bromide ions in one broad step to form copper oxide (Cu=O, 18%) as the final product.

### 3.10. Electrochemistry of complex 2

**Fig. 12:**
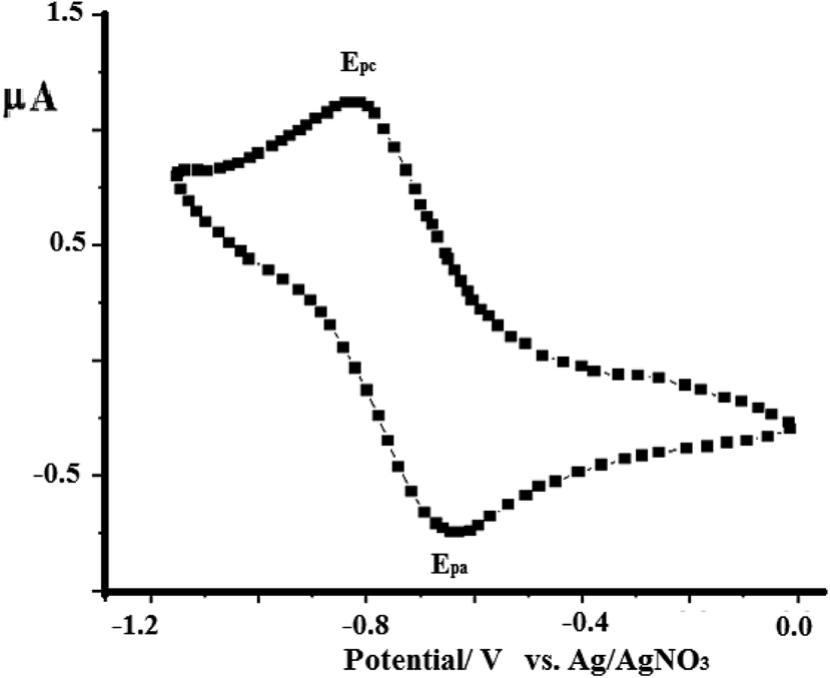
CV curve obtained for complex 1 dissolved in acetonitrile (1 × 10^−4^ M) at RT in the presence of 0.1 M TBAHF at a scan rate of 0.10 V s^−1^ using Ag/AgNO_3_ as reference electrode.

As a representative example, the electron-transfer conductance of complex 2 in acetonitrile was investigated using cyclic voltammetry, as shown in Fig. 12. The N_3_S_2_ ligand is electroinactive from 0 to −1.5 V, while complex 2 exhibits a one electron redox transfer process in this range. The electrochemical behavior observed at the Pt working electrode was *E*_1/2_ = −0.760 V, *i*_pa_/*i*_pc_ = 0.92, and Δ*E*_p_ =130 mV, and the plot of *i*_pc_*vs. v*^1/2^ was linear with slope = 0.991. All these parameters suggest that the Cu(i)/Cu(ii) redox process becomes quasi-reversible with responses at −650 and −780 mV (*vs.* Ag/AgNO_3_).

### 3.11. SEM and EDS

**Fig. 13:**
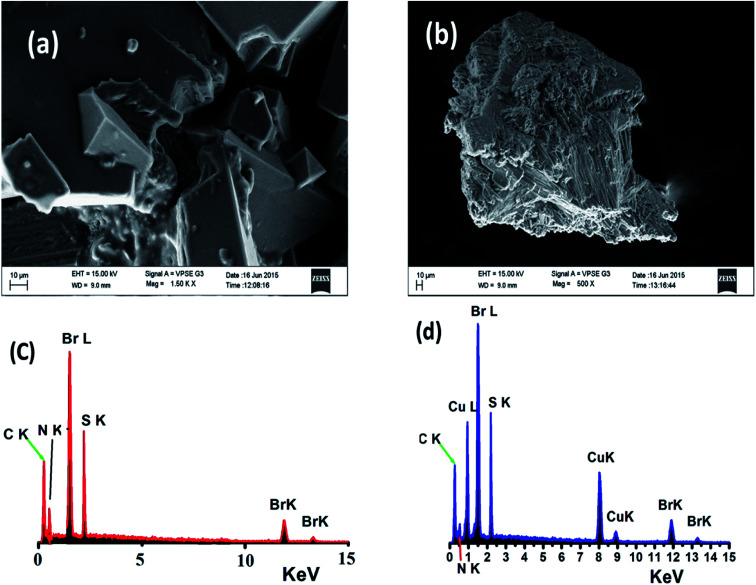
SEM images of (a) the N_3_S_2_ ligand and (b) complex 2, and EDS images of (c) the N_3_S_2_ ligand and (d) complex 2.

The surface morphologies of both the free N_3_S_2_ ligand and complex 2 were subjected to SEM and EDS. The SEM image of the free ligand displays a semi-square single phase with a block over block morphology with unequal boundaries and various micrometer volumes (Fig. 13a). The SEM image of complex 2 shows a different morphology with a smooth, homogeneous, and uniform rod-like morphology with various pore sizes (Fig. 13b).

Since the SEM image of the surface of the free N_3_S_2_ ligand was different than that of its corresponding complex, the change in the morphology of the ligand before and after its coordination to Cu(ii) confirmed the formation of the L–M complex and allowed us to differentiate the chemical composition of the N_3_S_2_ ligand and its complex. Therefore, the composition of the N_3_S_2_ ligand and its complex were determined using EDS, as shown in Fig. 13c and d, respectively. Comparison of the spectra indicated that the N_3_S_2_ ligand only contains C, N, S, and Br, while its complex contains C, N, S, Br, and Cu, which again confirmed the formation of the copper complex. The absence of O atoms in the ligand and complex indicates the stability of the compounds toward atmospheric O_2_. The absence of unknown peaks reflects the high purity of both the ligand and its complex.

### 3.12. CT-DNA binding affinity of complexes

#### 3.12.1. Absorption spectroscopy

Absorption spectroscopy is considered to be one of the most useful methods to evaluate the DNA binding affinity of a molecule.^35–40^ The affinity of complex 1 and 2 toward CT-DNA was investigated using the UV-Vis titration spectra recorded in Tris–HCl buffer solution. The UV-Vis spectra of the target compounds were expected to change upon drug-DNA binding. Bathochromic red shift hypochromism interactions are commonly observed due to strong π–π stacking (aromatic-DNA base pairs) and indicate intercalative binding interactions.^38–40^

**Fig. 14:**
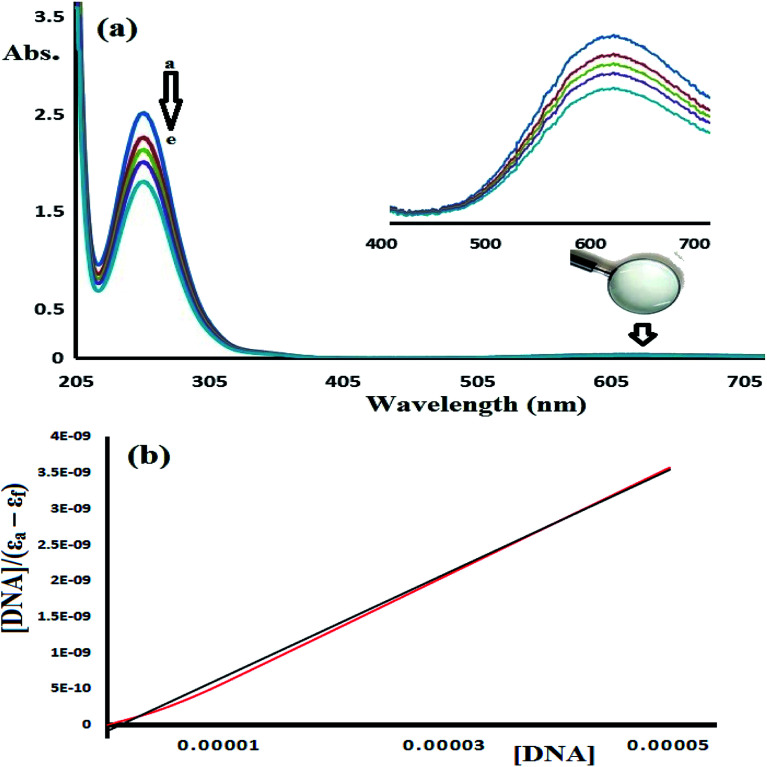
(a) UV-Vis spectra of complex 1 (5.0 × 10^−5^ M) recorded in the presence of 0, 1.0 × 10^−6^, 5.0 × 10^−6^, 1.0 × 10^−5^, and 5.0 × 10^−5^ M CT-DNA at RT (a → e). (b) Plot of [DNA]/(*ε*_a_ − *ε*_f_) *vs.* [DNA] observed at 250 nm used to determine *K*_b_.

**Fig. 15:**
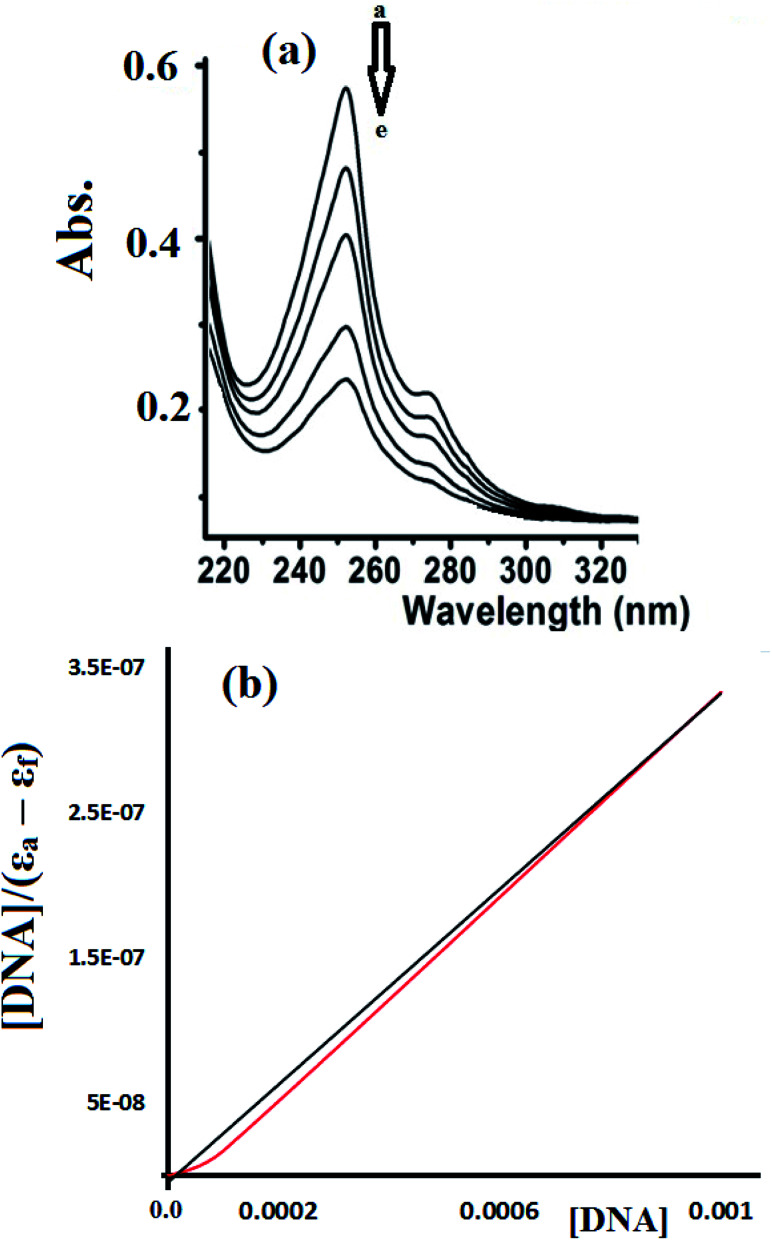
(a) UV-Vis spectra recorded for complex 2 (1.0 ×10^−5^ M) in the presence of 0, 1.0 × 10^−6^, 1.0 × 10^−5^, 1.0 × 10^−4^, and 1.0 × 10^−3^ M CT-DNA at RT (a → e). (b) Plot of [DNA]/(*ε*_a_ − *ε*_f_) *vs.* [DNA] observed at 255 nm used to determine the intrinsic binding constant (*K*_b_).

To estimate the binding ability of the Cu(ii) complexes, the intrinsic binding constant (*K*_b_) was evaluated by monitoring the changes in the absorbance spectra verses the CT-DNA concentration using the following equation.^35–38^[DNA]/(*ε*_a_ − *ε*_f_) = [DNA]/(*ε*_b_ − *ε*_f_) + 1/(*K*_b_ (*ε*_b_ − *ε*_f_)where [DNA] is the concentration of DNA in the base pairs and *ε*_f_, *ε*_a_, and *ε*_b_ are the free-, apparent-, and metal-bound complex extinction coefficients, respectively. *K*_b_ is the equilibrium binding constant (M^−1^) for complex 1 bound to DNA. When plotting [DNA]/(*ε*_a_ − *ε*_f_) *vs.* [DNA], *K*_b_ was obtained using the ratio of the slope to the intercept. The plot of [DNA]/(*ε*_a_ − *ε*_f_) *vs.* [DNA] was used to calculate and compare the *K*_b_ values obtained for complex 1 (Fig. 14) and complex 2 (Fig. 15).

Fig. 14 shows the UV-Vis spectra of CT-DNA-1. A high concentration of complex 1 (5 × 10^−5^ M) was used in this experiment in order to monitor the complex absorption behavior in both the UV and visible light regions during the DNA titration experiment. The two characteristic absorption peaks observed at 250 and 625 nm decrease in intensity upon the addition of CT-DNA at different concentrations, as shown in Fig. 14.

Fig. 15 shows the UV-Vis spectra recorded during the complex 2-CT-DNA binding titration experiment at 255 nm using a lower concentration of complex 2 (1 × 10^−5^ M).

The UV-abs. spectral titrations behavior of DNA with the Cu(ii) complexes were used to make the comparison between the desired complexes with the recent synthesized Schiff bases/Cu(ii) complexes. The small shift in Abs. wavelength and the intercalating binding constant (*K*_b_) values were used as a criterion in judging the [Cu(ii):DNA] binding strength as seen in Table 2.

**Table 2:** The [Cu(ii): DNA] binding strength data of recent Schiff bases/Cu(ii) complexes

No.	Complex no.	Shift in wavelength	*K*_b_	Ref.
1	Complexes 1–6	Bathochromic	2.41 × 10^4^–1.60 × 10^5^	41
2	CuCl_2_(SB)_2_	Hyperchromic	7.85 × 10^3^	42
3	Complex 2	Hyperchromic	3.14 × 10^3^	43
4	Complex 3	Hyperchromic	1.06 × 10^5^	44
5	CuLB	No shift	6.09 × 10^5^	45
6	Complex 1	Hyperchromic	3.34 × 10^4^	46
7	Cu(4a)	Bathochromic	9.05 × 10^5^	47
8	Complexes 1 and 2	Hyperchromic	3.20 × 10^5^ and 2.51 × 10^5^	This study

Depending on the results illustrated in Table 1, one can say that complexes 1 and 2 prepared in this study are classified as a good binder among their peers' complexes, as their *K*_b_ values are higher than all the complexes except complexes in entries 5 and 7. Such high activities can be attributed to the pentadentate coordination mode of N_3_S_2_ Schiff base ligand, which gave their Cu(ii) desired complexes an additional stability and opportunities to bind the DNA strings in different types and more binding modes, these features are equivalent to those previously observed for Cu(ii) complexes.^38–40^

#### 3.12.2. Viscosity

**Fig. 16:**
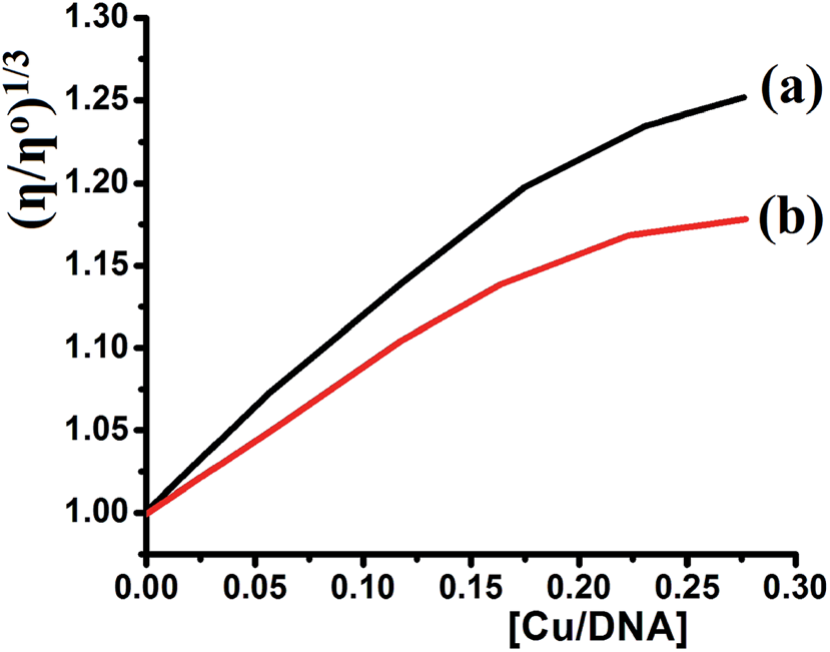
Effect of concentration of (a) complex 1 and (b) complex 2 [0, 2.5 × 10^−5^, 6.25 × 10^−5^, 8.75 × 10^−4^, 1.12 × 10^−4^, and 1.37 × 10^−4^ M) on relative viscosity of CT-DNA (5 × 10^−4^ M) at RT in Tris–HCl buffer.

To clarify the nature of the Cu(ii) complexes upon interaction with CT-DNA and determine which of the complexes is the better binder, the binding modes were investigated using viscosity measurements. The values of the relative specific viscosity were (*η*/*η*^o^)^1/3^ plotted against [complex]/[DNA] (Fig. 16). The viscosity of DNA was increased upon interaction with the complexes because they make the DNA longer.^37–40^ In this study using identical conditions, it was observed that increasing the complex concentration leads to an increase in the DNA viscosity (complex 1 > complex 2). Thus, complex 1 is a slightly better DNA binder than complex 2, which was in agreement with the DNA binding results.

## 4. Conclusions

A new Schiff base, (*E*)-*N*1-((5-bromothiophen-2-yl)methylene)-*N*2-(2-((*E*)-((5-bromothiophen-2-yl)methylene)amino)ethyl)ethane-1,2-diamine, was synthesized *via* the condensation of 5-bromothiophene-2-carbaldehyde and diethylenetriamine. The N_3_S_2_ ligand was characterized using spectroscopic and theoretical analyses, and the condensation reaction used in its synthesis was monitored through FT-IR spectroscopy. Water soluble square pyramid dicationic complexes with the general formula [Cu(N_3_S_2_)]X_2_ were formed because the N_3_S_2_ ligand acts as a pentadentate ligand. The TG results demonstrated the different thermal behavior observed between the free ligand and its complexes. The polarity of the solvent used plays a critical role in controlling the solvatochromatic behavior of the complexes. SEM and EDS supported the complexation of the N_3_S_2_ ligand to the Cu(ii) metal center. The complexes exhibited a one electron redox transfer process with negative voltages. Both the viscosity and absorption spectra showed that complex 1 is a better CT-DNA binder than complex 2 with *K*_b_ values of 3.20 × 10^5^ and 2.51 × 10^5^ M^−1^, respectively. The hypochromism interactions indicated and intercalative binding between the aromatic-DNA base pairs and the complexes theophine aromatic ring *via* π–π stacking interactions. The preliminary complex:DNA binding results observed in this study reflected the desired complexes as very good binder, which means that such compounds can be used as new chemotherapy.

## Conflicts of interest

The authors declare no conflict of interest.

## Supplementary Material
